# Berries as a Treatment for Obesity-Induced Inflammation: Evidence from Preclinical Models

**DOI:** 10.3390/nu13020334

**Published:** 2021-01-23

**Authors:** Hannah Land Lail, Rafaela G. Feresin, Dominique Hicks, Blakely Stone, Emily Price, Desiree Wanders

**Affiliations:** Department of Nutrition, Byrdine F. Lewis College of Nursing and Health Professions, Georgia State University, Atlanta, GA 30302, USA; hland2@student.gsu.edu (H.L.L.); rferesin@gsu.edu (R.G.F.); domin.hicks@gmail.com (D.H.); bstone9@student.gsu.edu (B.S.); eprice11@student.gsu.edu (E.P.)

**Keywords:** blackberries, raspberries, strawberries, blueberries, polyphenols, cytokines, adipose tissue, brain, microbiota, reactive oxygen species, oxidative stress

## Abstract

Inflammation that accompanies obesity is associated with the infiltration of metabolically active tissues by inflammatory immune cells. This propagates a chronic low-grade inflammation associated with increased signaling of common inflammatory pathways such as NF-κB and Toll-like receptor 4 (TLR4). Obesity-associated inflammation is linked to an increased risk of chronic diseases, including type 2 diabetes, cardiovascular disease, and cancer. Preclinical rodent and cell culture studies provide robust evidence that berries and their bioactive components have beneficial effects not only on inflammation, but also on biomarkers of many of these chronic diseases. Berries contain an abundance of bioactive compounds that have been shown to inhibit inflammation and to reduce reactive oxygen species. Therefore, berries represent an intriguing possibility for the treatment of obesity-induced inflammation and associated comorbidities. This review summarizes the anti-inflammatory properties of blackberries, blueberries, strawberries, and raspberries. This review highlights the anti-inflammatory mechanisms of berries and their bioactive components that have been elucidated through the use of preclinical models. The primary mechanisms mediating the anti-inflammatory effects of berries include a reduction in NF-κB signaling that may be secondary to reduced oxidative stress, a down-regulation of TLR4 signaling, and an increase in Nrf2.

## 1. Introduction

The prevalence of obesity among adults is increasing worldwide and is predicted to rise to 48.9% in the United States by 2030 [1]. Obesity is accompanied by chronic low-grade inflammation that affects most tissues in the body, including the liver [2], adipose tissue [3], brain [4], and pancreas [5], among others. The heightened inflammatory state of these metabolically important tissues has been postulated to increase the risk of developing type 2 diabetes [6,7], cardiovascular disease [8], and cancer [9].

Infiltration of adipose tissue, liver, pancreas, and other tissues by immune cells such as M1 macrophages [10] and T-cells [11] is a hallmark of obesity-associated inflammation. Obesity is also associated with increased circulating concentrations of inflammatory markers such as C-reactive protein (CRP) [12], interleukin-6 (IL-6) [13], and tumor necrosis factor (TNF)-α [13]. The transcription factor, nuclear factor kappa light chain enhancer of activated B cells (NF-κB) [14], and Toll-like receptor 4 (TLR4) [15] have been identified as important mediators propagating the effects of obesity on inflammation. These represent potential targets for novel treatment approaches for obesity-induced inflammation. In addition, in obesity, there is an imbalance in the ratio of pro-oxidant and antioxidant species, leading to oxidative stress, which can exacerbate inflammation and further cell damage [16].

Novel strategies to target obesity-associated inflammation have the potential to be effective in treating a number of chronic diseases. Among consumers, there is a growing interest in the use of foods with nutraceutical properties in the treatment and prevention of human diseases [17]. This consumer interest is reflected in trends seen in the growing dietary supplement market. Berries have been extensively studied for their anti-inflammatory properties that may translate to beneficial health outcomes.

There are several components of berries that can elicit beneficial health effects, including vitamins, minerals, fiber, and polyphenols. Different berries have different amounts and proportions of these phytonutrients, and the number and proportion of bioactive compounds will vary even within a type of berry due to genetic qualities, cultivation conditions, ripeness, and storage conditions [18]. Because they contain an abundance of bioactive compounds, berries have the potential to aid in the reduction in obesity-induced inflammation and related cardiometabolic dysfunction [18,19,20,21]. There are a wide variety of berries consumed globally, including blackberries, blueberries, strawberries, raspberries, cranberries, goji berries, sea buckthorn berries, and dog rose berries, among many others. Many of these berries have been demonstrated to exhibit beneficial effects on inflammation and biomarkers of health in humans and preclinical models [22,23,24,25,26]. Human feeding trials have demonstrated the efficacy of berries in improving blood glucose, insulin, and lipid concentrations and reducing circulating inflammatory markers [21,26]. Preclinical rodent and cell culture studies have elucidated mechanisms by which berries and berry-derived compounds elicit anti-inflammatory effects. A comprehensive review of the anti-inflammatory effects of berries in humans has been published [26]. The objective of this review is to critically evaluate, summarize, and compare the available literature reporting the effects of blackberries, blueberries, strawberries, and raspberries on inflammation. This review highlights the anti-inflammatory mechanisms of these berries and their bioactive components that have been elucidated through the use of rodent and cell culture models.

### Bioactive Components of Berries

Berries contain a number of bioactive compounds, among which are various types of polyphenolic compounds [18]. These compounds are increasingly being investigated for their potent antioxidant and anti-inflammatory abilities. They include compounds belonging to the flavonoid family, such as anthocyanidins (e.g., cyanidin, pelargonidin and peonidin glycosides), flavonols (e.g., kaempferol and quercetin glycosides), flavanols ((+)-catechin and (−)-epicatechin), flavanones (e.g., naringenin), and flavones (e.g., apigenin); phenolic acids (e.g., hydroxycynnamic and hydroxybenzoic acids); hydrolyzable (e.g., ellagitannins) and condensed (e.g., proanthocyanidins) tannins; and stilbenes (e.g., resveratrol). Anthocyanins are widely distributed in foods; thus, they are the most consumed flavonoid. They are water-soluble plant metabolites responsible for the vivid red, purple, and blue pigments found in many plants including berries [19]. These compounds are glycosylated polyhydroxy and polymethoxy derivatives of flavylium salts [27]. Cyanidin-3-glucoside (C3G) is considered to be the primary anthocyanin found in the edible portions of most plant foods [27] and is highly electron-deficient and can, therefore, scavenge free radicals and reactive oxygen species (ROS), giving it the highest oxygen radical absorbance capacity. Phenolic acids have an aromatic ring with one or more hydroxyl groups and a carboxylic function. They are found in abundance in berries and have tremendous antioxidant abilities as well as anticancer, antiviral, and antimicrobial benefits [27,28,29,30]. Proanthocyanidins are also found in berries and have powerful antioxidant abilities, and have been found to play a beneficial role in vascular relaxation in cardiovascular disease [31].

Six different anthocyanins were identified in a Hull blackberry extract [32], with the most prevalent being C3G. Blackberries also contain proanthocyanidins and ellagitannins, which are polymers of ellagic acid [18,33] and contribute to their ability to reduce oxidative stress and inflammation. Blueberries have the highest total polyphenolic content when compared to other berries [18]. Myricetin and quercetin glycosides are the major flavonols, while cyanidin, delphinidin and malvidin glycosides are examples of major anthocyanidins found in blueberries [18]. The most abundant single phytochemical present in blueberries is chlorogenic acid, which has been shown to aid in lipid metabolism [19].

Strawberries contain several polyphenols known to exhibit anti-inflammatory effects. The total phenolic content of strawberries is similar to blackberries and raspberries, but lower than blueberries [18]. Compared to these berries, the anthocyanin content is lowest in strawberries [18]. The primary anthocyanidins in strawberries are pelargonidin 3-glucoside and C3G [34]. Strawberries are also rich in ellagitannins. Raspberries contain similar amounts of ascorbic acid as strawberries and blackberries [18]. The major phenolic compounds in raspberries include flavonols such as kaempferol glycosides and quercetin, anthocyanidins such as cyanidin and pelargonidin glycosides, phenolic acids such as ellagic acid, and hydrolyzable tannins such as ellagitannins, among many others [18]. Recently, raspberry ketone (4-(4-hydroxyphenyl butan-2-one)), an aromatic compound responsible for providing fragrance and flavoring, has been of interest for its potential anti-obesity and anti-inflammatory effects. However, the raspberry ketones used in many of these studies are synthetic supplements, not naturally derived compounds. The polyphenolic compounds found in berries, and specifically berry anthocyanidins, have potent antioxidant and anti-inflammatory properties that may prevent obesity-related disorders, including type 2 diabetes [32,35,36]. A detailed report of the bioactive components found in blackberries, blueberries, strawberries, and raspberries has been previously described [18].

## 2. Anti-Inflammatory Effects of Blackberries

### 2.1. Obesity-Induced Inflammation

Several studies have been conducted in preclinical rodent models to determine the mechanism by which blackberries or their bioactive components affect obesity-induced inflammation. Many of these studies found that blackberries or their bioactive components not only reduce inflammation, but also improve other markers of metabolic health, including increasing insulin sensitivity and reducing adiposity. To investigate the anti-obesity effect of blackberry anthocyanins, Wu et al. placed male C57BL/6J mice on a high-fat diet (HFD) with 45% calories from fat for 12 weeks [37]. The HFD induced inflammation and oxidative stress in the mice. Mice were assigned to an HFD plus supplementation of purified anthocyanins from either blackberries (93.32% C3G and 6.68% peonidin-3-glucoside) or blueberries (51.24% C3G, 42.31% cyanidin-3-rutinoside, and 6.91% peonidin-3-glucoside). Additionally, three control groups were administered either an HFD, a low-fat diet (LFD), or an HFD supplemented with orlistat, a pharmacological anti-obesity compound, for 12 weeks. Compared to the HFD-control group, the mice supplemented with blackberry anthocyanins at a dose of 200 mg/kg body weight for 12 weeks gained 40.5% less body weight. The reduction in body weight seen in the mice consuming the blackberry anthocyanins was comparable to the mice receiving orlistat. Blackberry anthocyanins also attenuated the HFD-induced increases in epididymal fat mass, serum and hepatic triglycerides concentrations, and serum total cholesterol concentrations. Consuming either blackberry or blueberry anthocyanins decreased the expression of several inflammatory markers, including NF-κB, IL-6, and TNF-α in the liver, with blueberry anthocyanins having a stronger effect [37]. This suggests that blackberry anthocyanins inhibit obesity-induced hepatic inflammation, possibly through downregulation of NF-κB signaling. Consumption of blackberry anthocyanins suppressed obesity-induced oxidative stress. The reduction in markers of oxidative stress in the liver and serum were likely due to the increase in activities of the antioxidant enzymes superoxide dismutase (SOD) and glutathione peroxidase (GPx) in the liver [37].

Similarly, another study conducted in C57BL/6J mice reported that the phenolic compounds isolated from a fermented blueberry-blackberry beverage mitigated HFD-induced metabolic dysfunction. The phenolic extract at daily doses of 9 or 18.9 mg/kg body weight attenuated increases in adipose tissue mass, plasma triglycerides, plasma cholesterol, and fasting blood glucose concentrations [38]. In this study, however, the 60% fat diet for 11 weeks failed to induce significant inflammation in the pancreas, adipose tissue, or liver. Therefore, the researchers could not draw any conclusions about the anti-inflammatory effects of the phenolic extract from the blueberry-blackberry beverage [38].

In ovariectomized rats, an animal model for human menopause, ovariectomy increased body weight, and a 100-day freeze-dried blackberry powder treatment (10% of the diet) attenuated the increase in body weight [39]. The 10% blackberry diet also reduced mRNA expression of NF-κB and cyclooxygenase-2 (COX-2) in the liver [39]. While these data point to a reduction in inflammation, there was no report of the effect of blackberry consumption on tissue or circulating cytokines and markers of inflammation in other tissues. Furthermore, a reduction in mRNA expression of NF-κB and COX-2 do not necessarily translate to a reduction in their activities.

The effect of blackberries on metabolic parameters is somewhat inconsistent. One study reported that mice fed an HFD supplemented with blackberry (6.3% of the diet) for 12 weeks showed no significant differences in their body weight, liver weight, epididymal adipose tissue weight, hepatic triglycerides, or total cholesterol when compared to the HFD-control group [36]. However, plasma triglycerides remained significantly lower in the blackberry supplemented group. Blackberry consumption did not affect gene expression of inflammatory markers (TNF-α, IL-6, and MCP-1) or crown-like structure formation in adipose tissue. There are some important differences between this study and the previous studies that found the effects of bioactive components from blackberries on obesity-induced metabolic parameters. First, while the other studies used diets with a higher percentage of energy from fat, the HFD used in this study contained only 35% of kcal from fat. Second, the mice used in these studies were considerably older (12 weeks of age at the start of the study) than the mice in the other studies, which were ~3 weeks of age. Lastly, the administration of the berries differed. While the previously discussed studies used extracts of bioactive components from berries, this study fed the animals freeze-dried berry powder mixed in the diet in an amount that could feasibly be consumed when considering translation to human consumption (6.3% of the diet by weight) [36].

Additionally, another study assessing the effects of different berries on weight gain and inflammation in C57BL/6J mice also saw no significant improvements in any metabolic markers of mice supplemented with blackberries (20% of the diet by weight for 13 weeks) compared to the HFD-control group [35]. Similar to the previous study that also saw no effect of blackberry supplementation on HFD-induced weight gain and metabolic parameters, this study administered the berries as a freeze-dried powder, not a bioactive component extract. Due to the variability among the results of these studies, additional investigation is warranted to determine whether blackberry supplementation exhibits protective effects in an in vivo model of obesity. Subsequent investigation into the mechanism by which blackberries may ameliorate the harmful effects of obesity will be important.

### 2.2. Macrophage and Adipocyte Inflammation

HFD-induced obesity is associated with the infiltration of macrophages into the adipose tissue, which accounts for a higher concentration of proinflammatory cytokines such as TNF-α and IL-6 in the adipose tissue, as well as an increase in inducible nitric oxide synthase (iNOS) expression [40,41]. Macrophages are recruited to the adipose tissue, in part, in response to elevated monocyte chemoattractant protein-1 (MCP-1) produced from adipocytes during obesity [36,40,42]. Once inflammatory macrophages are present in tissues, they contribute to the inflammatory milieu by producing inflammatory cytokines. Several studies have investigated whether blackberries impact these pathways. Blackberry anthocyanin extract (0–20 µg/mL) has been shown to prevent LPS-induced inflammatory cytokine production from RAW 264.7 macrophages [43]. Cells were pretreated with the anthocyanin extracts for 12 h. Then, 100 ng/mL of LPS was added for 3 h or 24 h. The mechanism by which blackberry anthocyanins suppressed LPS-induced increases in IL-1β and TNF-α production from macrophages was by suppressing NF-κB signaling [43]. Additionally, Nrf2 was shown to play a role in the ability of blackberry anthocyanins to suppress oxidative stress, but not inflammation [43]. Treatment of bone marrow-derived macrophages from wild-type mice with blackberry anthocyanins (20 µg/mL) suppressed ROS levels and IL-1β mRNA. However, the ability of the berries to reduce cellular ROS concentrations was attenuated in bone marrow-derived macrophages isolated from *Nrf2* knockout mice. These data suggest that NRF2 plays a role in the ability of blackberry anthocyanins to reduce oxidative stress, but not in their ability to reduce inflammation [43].

Similarly, a 2014 study found that anthocyanins extracted from a blueberry-blackberry dealcoholized fermented beverage reduced inflammation in RAW 264.7 macrophages and 3T3-L1 adipocytes [44]. Pretreating the macrophages with anthocyanin-enriched fractions (100 μM C3G equivalents) for one hour suppressed the LPS-induced secretion of nitric oxide (NO) and TNF-α [44]. The LPS stimulation was performed at a dose of 1 μg/mL of LPS for 24 h. The mechanism by which berries suppressed inflammation was likely due to the inhibition of NF-κB signaling, as all anthocyanin blends effectively decreased the level of expression of the p65 subunit of NF-κB. The anthocyanin extracts were also able to increase the expression, but not secretion, of the anti-inflammatory, insulin-sensitizing adipokine, adiponectin, in differentiated 3T3-L1 adipocytes incubated for 24 h in the presence of conditioned media from LPS-stimulated macrophages [44]. In another study, Johnson et al. treated RAW 264.7 macrophages with 50 and 100 µM of blackberry anthocyanins or proanthocyanidins in addition to LPS (1 µg/mL) for 24 h. They found that, at both doses, anthocyanins but not proanthocyanidins suppressed LPS-induced phosphorylation of the p65 subunit of NF-κB [33]. Furthermore, treatment with 100 µM of blackberry proanthocyanidins inhibited iNOS expression, while treatment with 100 µM of blackberry anthocyanins decreased NO levels. Together, these findings suggest that obesity-induced inflammation, characterized by the infiltration of macrophages to adipose tissue and the excessive production of NO and inflammatory cytokines in adipose tissue, may be attenuated by the administration of anthocyanins extracted from blackberries. Most of the studies implicated a down-regulation of NF-κB signaling as the primary mechanism mediating the anti-inflammatory effects of blackberries.

Other pathways have also been implicated in mediating the anti-inflammatory properties of blackberries. For example, blackberry extract was shown to suppress ultraviolet-B (UVB) radiation-induced inflammation in the skin of hairless mice, likely through a reduction in COX-2, prostaglandin E_2_ (PGE_2_), and iNOS [45]. Topical treatments of 10% or 20% of blackberry extract in acetone to mouse dorsal skin were performed the day before UVB exposure (100 mJ/cm^2^ of UVB, 3 times per week for 10 weeks). This study also found that blackberry extract inhibited the UVB-induced translocation of NF-κB to the nucleus. In addition, the UVB-induced activation of IKKα and the degradation of IκBα were both inhibited in mice who received topical application of blackberry extract [45]. This is relevant because activated IKKα works to degrade IκBα by phosphorylating its serine residues, enabling the activation of NF-κB.

Other studies have also shown that blackberry anthocyanins suppress LPS-induced COX-2 expression in RAW 264.7 macrophages [33]. Anthocyanins and proanthocyanidins extracted from blueberry-blackberry wine blends decreased LPS-induced COX-2 and iNOS expression [33]. It is worth noting that mixtures containing a higher concentration of blackberry compounds relative to blueberry compounds had a greater effect on COX-2 than on iNOS [33]. The downregulation of iNOS expression induced by blackberry anthocyanins was accompanied by reduced NO production from RAW 264.7 macrophages [33]. Therefore, it is likely that blackberries reduce inflammation through different mechanisms, including downregulation of NF-κB, iNOS, COX-2, and PGE_2_ signaling. Together, these findings indicate that blackberries or their extracts may constitute an effective treatment or preventative for inflammation induced by different stimuli, including HFD and obesity, and that blackberries likely target multiple anti-inflammatory pathways.

### 2.3. Dysbiosis and Neuroinflammation

Obesity has been linked to neuroinflammation, mental health concerns, and changes in cognitive behavior [46,47]. An HFD can induce alterations in the composition of the gut microbiota, known as dysbiosis [40,46]. In turn, HFD-induced dysbiosis is reported to play a role in neuroinflammation [48]. Anthocyanins from blackberries may be able to reduce neuroinflammation by attenuating the HFD-induced alteration of the gut microbiota [49,50,51]. To investigate the effects of blackberry anthocyanin-rich extract (25 mg/kg body weight/day) on gut health and neuroinflammation, four groups of Wistar rats were given a standard diet, an HFD (45% fat), a standard diet supplemented with blackberry extract, or an HFD supplemented with blackberry extract for 17 weeks [46]. Gut microbiota composition was analyzed at the phylum and genus levels. Upon analysis, blackberry extract was found to partly prevent HFD-induced modification and loss of diversity in the gut microbiota. Notably, blackberry extract countered the increase in the genus *Rumminococcus* and the decrease in the genus *Sporobacter* observed in HFD-control rats compared to rats who received the standard diet. However, this effect was not statistically significant. Blackberry extract also increased the prevalence of *Pseudoflavonifractor* in animals fed a standard diet compared to the standard diet control group and increased the presence of *Oscillobacter* independent of the fat content of the diet. The presence of *Pseudoflavonifractor* and *Sporobacter* was negatively correlated with hippocampal TCK-1 (CXCL7), a neuroinflammatory marker [46]. This is consistent with an earlier study by the same group concluding that chronic blackberry extract consumption (25 mg/kg body weight/day) for 17 weeks decreased the expression of TCK-1 in the hippocampus of Wistar rats fed an HFD (45% fat) [49]. These findings suggest that blackberry extract may protect against neuroinflammation due to its ability to attenuate HFD-induced dysbiosis. To elucidate the underlying mechanism responsible for this beneficial effect, fecal samples from the rats were analyzed for LPS, which triggers an inflammatory response through activation of TLR4 [46]. There was a trend for the HFD to increase fecal LPS levels, and for blackberry extract to attenuate this increase. However, these results were not statistically significant [46]. Fecal concentrations of tryptophan were significantly lower in rats that received blackberry extract supplementation (25 mg/kg body weight/day for 17 weeks). Fecal tryptophan concentrations were positively correlated with TCK-1 expression in the hippocampus [46]. The tryptophan pathway can produce metabolites such as kynurenic acid, which are thought to have anti-inflammatory properties and play a role in central nervous system excitation [52,53]. This suggests that tryptophan metabolism may mediate the protective effects of blackberry extract against neuroinflammation. Additional studies proposed that HFDs are sufficient to cause obesity-related inflammation by altering gut microbiota composition, increasing intestinal permeability, and inducing TLR4 signaling [40,47,54]. Together, these findings suggest that blackberries may prevent HFD-induced activation of the proinflammatory TLR4 pathway by inhibiting an alteration in the composition of gut microbiota and improving intestinal permeabilization.

The series of articles presented in this review tended to find beneficial effects of blackberries or their bioactive compounds in different models of inflammation. However, many of these studies did not identify which specific compounds extracted from blackberries are responsible for the anti-inflammatory properties, nor did they entirely elucidate their underlying mechanisms. Anthocyanins, C3G in particular, seem to be the most abundantly researched bioactive component. This may be because they are considered the most effective bioactive compounds for the prevention of oxidative stress and inflammation. Furthermore, in addition to C3G being the most prevalent anthocyanin found in blackberries, blackberries also have the highest amount of C3G compared to other berries. Differences in anthocyanin composition may result in different anti-inflammatory effects and mechanisms of action. Overall, blackberries show great potential for the treatment of obesity-induced inflammation, but further investigation is needed to identify specific components and their underlying mechanisms.

## 3. Anti-Inflammatory Effects of Blueberries

### 3.1. Obesity-Induced Inflammation

Several studies have been conducted in preclinical rodent models to determine the mechanism by which blueberries or their bioactive components affect obesity-induced inflammation. Many of these studies have found that blueberries or their extracts are effective at reducing inflammation and concomitantly improving other biomarkers of metabolic health. For instance, supplementing an HFD (45% fat) with blueberry anthocyanins (200 mg/kg food; equivalent to ~2 mg/kg body weight in humans) for 12 weeks attenuated HFD-induced increases in body weight and adiposity in C57BL/6 mice [37]. Blueberry anthocyanins also reduced HFD-induced increases in total cholesterol, serum triglycerides, and circulating and hepatic malondialdehyde (MDA; a marker of oxidative stress) concentrations [37]. The blueberry anthocyanins reduced the expression of IL-6, TNF-α, and NF-κB expression in the liver of HFD-fed mice [37]. These data indicate that blueberry anthocyanins inhibit obesity-induced hepatic inflammation, possibly through downregulation of NF-κB signaling. The ability of blueberry anthocyanins to reduce MDA concentrations is likely due to increases in hepatic and serum SOD concentrations and GPx activity [37].

Similarly, another study found that supplementing an HFD (45% fat) with Nordic wild blueberries (10% by weight) for 12 weeks reduced weight gain in C57BL/6 mice [55]. They also reported beneficial changes in the status of serum concentrations of the proinflammatory markers, IL-1β, IL-2, TNF-α, and GM-CSF with decreases trending toward control levels. There was as a significant reduction in serum MCP-1 concentrations of mice supplemented with both 5% and 10% freeze-dried blueberries when compared to control [55]. This study did not report on any potential mechanisms mediating the anti-obesity and anti-inflammatory effects of the berries.

The results of studies investigating the effects of blueberries on body weight are somewhat inconsistent. For example, a study using obese C57BL/6 mice found that supplementing the HFD (35% fat) with 5% blueberry powder by weight (470 mg C3G equivalents/kg diet) for 12 weeks had no effect on body weight, adiposity, or any markers of inflammation or oxidative stress in liver, adipose tissue, or plasma [36]. Another study also using C57BL/6 mice fed an HFD (60% fat) found no effect of 8 weeks of blueberry supplementation on body weight or adiposity [56]. This study only supplemented the HFD with 4% blueberry powder by weight, compared to the previously mentioned study, which saw significant effects on body weight with 10% blueberry powder [55]. Despite not reducing body weight or adiposity, the addition of 4% blueberry powder significantly reduced the presence of CD11c^+^ (M1; inflammatory) macrophages and TNF-*α* expression in adipose tissue [56]. These anti-inflammatory effects were accompanied by a significant improvement in insulin sensitivity [56]. This study did not report any mechanisms by which blueberries exert these effects. However, it is clear that blueberries exert their benefits independent of reductions in body weight or adiposity. The berries have direct anti-inflammatory and insulin-sensitizing effects. This is also made clear by use of cell culture models which show the direct effects of blueberry extracts or bioactive compounds on inflammation and insulin sensitivity.

Similarly, a study that fed obese Zucker rats a control chow diet or a diet supplemented with 2% blueberry powder for 15 weeks found that while blueberry consumption did not affect body weight, it did improve glucose tolerance and attenuate renal oxidative stress and inflammation [57]. Blueberry consumption reduced the expression of IL-1β and IL-18, both of which are downstream targets of the TLR4 pathway. This study also identified a reduction in MAPK phosphorylation, TLR4 gene and protein expression, and NF-κB activity as potentially mediating the blueberry-induced reduction in inflammation in the kidney [57]. The rats fed the blueberry-enriched diet had increased expression of Nrf2 and increased presence of the antioxidant enzymes, SOD and catalase, which explains the mechanism by which blueberries reduced renal oxidative stress [57].

In another study, lean and obese Zucker rats were fed a diet supplemented with 8% wild blueberry powder for 8 weeks [58]. Obese Zucker rats had elevated circulating concentrations of IL-6, TNF-α, and CRP compared to lean rats. Despite not affecting body weight, supplementation with wild blueberry significantly reduced concentrations of these inflammatory markers in the plasma of obese rats. Furthermore, wild blueberry supplementation reduced hepatic expression of NF-κB, IL-6, TNF-α, and CRP in obese Zucker rats. In adipose tissue, wild blueberry supplementation reduced expression of NF-κB, IL-6, and TNF-α in obese Zucker rats [58]. While this study suggested that reduced NF-κB signaling may be responsible for the reduction in inflammatory markers in the liver, adipose tissue, and serum, further investigation is needed, as only NF-κB mRNA was measured.

At higher concentrations, anthocyanins from blueberries were effective at reducing IL-1β and TNF-α expression in RAW 264.7 macrophages treated with 20 μg/mL of blueberry extract for 3 h [43]. This reduction was likely due to decreased NF-κB signaling, as the blueberry anthocyanins suppressed nuclear expression of NF-κB p-65. Furthermore, blueberry anthocyanins reduced cellular ROS concentrations, NADPH oxidase (Nox)1 expression, and IL-1β expression in bone-marrow derived macrophages. The ability of blueberry anthocyanins to reduce cellular ROS was blunted in macrophages isolated from Nrf2 knockout mice, while their ability to reduce IL-1β and Nox1 remained intact. These data suggest that blueberry anthocyanins reduce oxidative stress in macrophages in an Nrf2-dependent manner, while they reduce inflammation independent of Nrf2 [43].

### 3.2. Retinal Damage

Obesity is linked to retinal diseases such as age-related cataracts, glaucoma, age-related maculopathy, and diabetic retinopathy [59]. Boosting the body’s antioxidant status has been suggested as a possible preventative measure against the development of retinal oxidative damage [60]. One study sought to explore the protective and functional role of 10 μg/mL blueberry anthocyanin extract and its predominant constituents, malvidin (Mv), malvidin-3-glucoside and malvidin-3-galactoside, on high glucose-induced injury in human retinal capillary endothelial cells [61]. Following both 24 and 48 h of treatment, the results show that blueberry anthocyanin extract and its constituents significantly enhanced cell viability, decreased ROS, and increased catalase and SOD activity in the cells. Moreover, blueberry anthocyanin extract initiated favorable alterations in NO levels and intercellular adhesion molecule-1 (ICAM-1), further reducing oxidative stress [61]. In a similar study using human retinal pigment epithelial cells, 5 μg/mL blueberry anthocyanin extracts significantly reduced hydrogen peroxide-induced oxidative stress by increasing SOD, catalase, and GPx after just 2 h of treatment [62].

To determine whether blueberries can protect against diabetes-induced oxidative stress and inflammation rat retinas in vivo, male rats were injected with streptozotocin to induce type 1 diabetes [60]. Blueberry anthocyanins were orally administered at 20, 40, and 80 mg/kg body weight for approximately 12 weeks. Blueberry anthocyanins were found to upregulate the antioxidant capacity of the rat retina, increase serum glutathione and GPx activity, and decrease ROS levels, suggesting that blueberry anthocyanins can protect retinal cells from diabetes-induced oxidative stress. Furthermore, blueberry anthocyanins reduced the streptozotocin-induced increase in retinal IL-1β in a dose-dependent manner. The mechanism by which blueberry anthocyanins reduced oxidative stress and inflammation in the retina was likely through an increase in NRF2 signaling, as blueberry anthocyanins increased nuclear NRF2 protein expression in the retina in a dose-dependent manner [60]. Many studies further supported these findings in various in vivo and in vitro models with consistent results indicating that blueberries decrease ROS activity and increase antioxidant activity levels [63]. Together, these findings suggest a possible role for blueberries in the prevention and treatment of obesity-induced retinal damage.

### 3.3. Vascular Endothelial Inflammation

Atherosclerosis is the primary cause of cardiovascular disease. Atherosclerosis is characterized by inflammation involving endothelial cell activation and secretion of adhesion molecules such as vascular cell adhesion molecule-1 (VCAM-1) and mobilization of monocytes to the arterial wall [64]. Once in the tissue, the monocytes differentiate into macrophages, which take up lipids and become foam cells, starting the formation of atherosclerotic plaques. Both diabetes and obesity increase the risk of cardiovascular disease and can lead to chronic inflammation of vascular endothelial cells [65,66]. The endothelial glycocalyx is made up of glycosaminoglycans (GAGs), or proteoglycans with GAG side chains, which normally function to protect the lumen from inflammation and sort incoming nutrients [67]. In diabetes, the vascular endothelium is bound by excess monocytes and is unable to function correctly as a protective barrier [68,69]. The net result is severely compromised GAGs, which lead to further increased inflammation and cell damage [68,69]. Recent studies have shown the potential for blueberries, particularly their anthocyanin components, as a treatment for vascular complications [70,71,72]. It is hypothesized that postprandial circulating blueberry metabolites are responsible for the fruits’ vascular benefits [73]. A recent study using human vascular endothelial cells derived from either healthy donors or donors with diabetes sought to determine if blueberry metabolites could improve endothelial inflammation by restoring the cell surface GAGs [74]. To test if blueberry metabolites could mitigate inflammation, aortic endothelial cells were treated with either blueberry metabolites (75 nM vanillic acid-4-sulfate, 75 nM isovanillic acid-3-sulfate, 700 nM benzoic acid-4-sulfate, 3 μM hydroxyl hippuric acid, and 5 μM hippuric acid) or vehicle for three days. As predicted, blueberry metabolites suppressed monocyte binding and reduced expression of the inflammatory markers, IL-8 and VCAM-1 in cells isolated from participants with diabetes [74]. They further discovered that the blueberry metabolites restored GAGs in the endothelial cells of participants with diabetes when compared to vehicle, suggesting that restoration of GAGs is responsible for the reduction in endothelial inflammation [74]. Additional studies supported the potential for blueberry metabolites as a mediator in the reduction in monocyte adhesion and inflammation in vascular endothelial cells [75,76,77].

### 3.4. Dysbiosis and Inflammation

As previously mentioned, emerging evidence suggests that obesity-associated inflammation may originate from an alteration in the microbiota of the gastrointestinal tract [78]. Blueberries have become of interest in the treatment of many diseases because of their rich phenolic content, which has powerful antioxidant and anti-inflammatory properties, and their excellent source of fermentable plant fiber [18,79]. These compounds have the potential to counteract the effects of obesity on gut microbiota. Blueberry components, such as anthocyanins, can reach the colon after ingestion, where they interact with the gut microbiota [80]. Not surprisingly, supplementing an HFD (45% fat) with 10% blueberry powder altered the composition of the gut microbiota in Wistar rats [81]. There was a decrease in the ratio of *Firmicutes* to *Bacteroidetes* following blueberry supplementation. In addition, dietary blueberry supplementation restored gastrointestinal integrity and ileal villus length when compared to the HFD alone. These changes to the gut microbiota were accompanied by reduced expression of IL-1β, TNF-α, and CD11d in adipose tissue, despite the blueberry supplementation not affecting body weight or adiposity. However, the alterations in gut microbiota and reductions in adipose tissue inflammation did not translate to a significant improvement in glucose tolerance [81]. The mechanisms mediating the anti-inflammatory effects of blueberries may involve a reduction in both TLR4 and NF-κB signaling, as circulating LPS concentrations and adipose tissue NF-κB phosphorylation were both reduced in the blueberry-supplemented group [81]. Another study found that supplementing an HFD with 200 mg/kg body weight blueberry polyphenol extracts for 12 weeks reduces energy intake and body weight in mice, which was accompanied by alterations in the gut microbiota [82]. However, inflammation was not evaluated in this study [82]. Evidence supports the theory that the HFD promotes unfavorable alterations in the gut microbiota, and the resulting inflammation is responsible for hyperphagia and obesity [78]. Therefore, if blueberry supplementation can beneficially alter the composition of the gut microbiota, restore intestinal anatomy, and reduce inflammation, it could be considered as a therapeutic strategy in the treatment of obesity.

### 3.5. T-Cell Immune Function and Inflammation

Emerging evidence suggests that one consequence of obesity is the dysregulation of cell-mediated immune responses, which contributes to compromised immune defense, a leading cause of infection in people with obesity [83]. C57BL/6 mice are a commonly used mouse model of diet-induced obesity. C57BL/6 mice fed a HFD develop increased adiposity, inflammation, and insulin resistance. Their obesity-induced inflammatory state is marked by elevated cytotoxic T-cell (Tc) and CD8^+^ cells and reduced presence of CD4^+^ helper and regulatory T-cells in adipose tissue, resulting in decreased adaptive immune response [84]. Because of their anti-inflammatory and antioxidant properties, blueberries may be effective in improving immune response in obesity by restoring T-cell-mediated function [85].

Supplementation of an HFD (45% fat) with freeze-dried wild blueberries (10% of the diet by weight) for 7 weeks reduced body weight and serum MCP-1 concentrations in C57BL/6 mice [55]. These changes were accompanied by a reduced percentage of Th1 cells in the spleen. Notably, wild blueberry supplementation at 5% of the diet by weight failed to induce similar changes [55]. Interestingly, another study found that supplementation of an HFD (60% fat) with blueberry powder at 4% of the diet by weight for 12 weeks increased T-cell proliferation and restored innate immune response from isolated splenocytes [85]. These results support the notion that T-cells play a role in the inflammatory and immune response processes in obesity and may be an important target of supplemental blueberries.

## 4. Anti-Inflammatory Effects of Strawberries

### 4.1. Obesity-Induced Inflammation

Given the palatability and bioactive compound profile of strawberries, they have the potential to serve as a functional food for the treatment of obesity-induced inflammation. In fact, when strawberry powder enriched with the major strawberry anthocyanin, pelargonidin 3-glucoside, was given to rats with diet-induced metabolic syndrome for six weeks, the strawberry powder reduced body weight and adipose tissue mass [34]. It is possible that these effects on body weight and composition were due to a significant reduction in food intake [34]. While circulating concentrations of CRP were unchanged, histological analysis showed that addition of the pelargonidin 3-glucoside-enriched strawberry powder to the diet mitigated some of the of the detrimental effects of metabolic syndrome on the liver. Specifically, the strawberry powder reduced hepatic lipid accumulation and inflammatory cell infiltration [34]. Strawberry powder also reduced inflammatory cell infiltration and fibrosis in the left ventricle of the heart. Mechanisms mediating these anti-inflammatory effects were not reported. The reduction in inflammatory cell infiltration in the liver and left ventricle was accompanied by significant reductions in diastolic stiffness and systolic blood pressure.

In another study, a combination of freeze-dried strawberry-blueberry powder (5:1) was added to the diet (6% of the diet by weight) of Wistar rats fed a high-fat, high-sucrose diet [86]. Despite increasing energy intake, eight weeks of strawberry-blueberry powder reduced body and visceral adipose tissue weight. This was accompanied by an improvement in glucose tolerance. While the strawberry-blueberry powder had no effect on expression of inflammatory markers in the adipose tissue, it did reduce circulating MCP-1 concentrations [86]. Since this supplementation was provided as a mixture of strawberries and blueberries, the effects cannot be attributed to one berry. However, the presence of strawberries in the mixture outnumbered blueberries 5:1.

Adding 2.6% strawberry powder (equivalent to ~one human serving of strawberries daily) to the diet had little effect on inflammation in mice consuming low-fat or high-fat diets [87]. Interestingly, the strawberry powder significantly reduced circulating CRP concentrations in mice consuming the low-fat, but not the high-fat diet. When splenocytes were isolated from the mice, it was evident that strawberry consumption also had no effect on splenocyte production of IL-6, IL-1β or TNF-α in response to LPS [87]. The lack of robust effects of strawberry powder in this study may be due to the relatively low, though physiologically relevant, amount of strawberries incorporated into the diet. The lack of effects of strawberries was likely not due to insufficient feeding time, as the mice were fed the diets for 24 weeks. It would have been interesting to see whether strawberry consumption affected inflammation localized to different metabolic tissues, as the authors noted a reduction in non-fasting blood glucose concentrations [87]. Unfortunately, mechanistic studies examining anti-inflammatory properties of strawberries in preclinical animal models is limited. A small number of studies have shown the efficacy of strawberries in suppressing inflammatory markers in humans [26], but additional investigations into the protective effects of strawberries against inflammation in vivo are warranted.

### 4.2. LPS-Induced Inflammation

High-fat diets can lead to a chronic elevation in circulating LPS concentrations [88], and high LPS has been associated with obesity in humans [89]. Interestingly, pretreatment of human dermal fibroblasts for 24 h with strawberry extract (50–1000 µg/mL) suppressed LPS-induced increases in intracellular ROS. Strawberry extracts at 100 µg/mL and 1000 µg/mL reduced LPS-induced increases in nitrite in the fibroblasts [90]. This ability of strawberry extracts to inhibit LPS signaling may explain their anti-inflammatory properties. Ellagic acid has been identified as one of the bioactive compounds in strawberries that is likely to mediate their anti-inflammatory properties [91]. Treatment of RAW 264.7 macrophages with strawberry extracts rich in ellagic acid or with ellagic acid alone (0.3 and 1.0 µM) reduced LPS-induced increases in iNOS, TNF-α, and IL-1β [91]. Evidence suggests that the mechanism by which the ellagic acid-rich strawberry extract suppressed LPS-induced inflammation is by inhibiting the NF-κB pathway. Treating the macrophages with this strawberry extract prevented the LPS-induced reduction in IκBα, an inhibitor of the NF-κB signaling pathway [91]. Another study found that pretreating RAW 264.7 macrophages with strawberry extract (100 µg/mL) for 24 h reduced LPS-induced increases in ROS, TNF-α, IL-1β, and IL-6 [92]. While NF-κB expression was not affected by the strawberry extract, pIκBα and iNOS expression were significantly reduced. Furthermore, treatment with the strawberry extract increased expression of Nrf2, but not SOD, catalase, or heme oxygenase-1 (HO-1) [92]. Together, these data suggest that strawberry extracts exert direct anti-inflammatory and antioxidant effects on cells, likely mediated through upregulation of the Nrf2 pathway and downregulation of the NF-κB pathway.

### 4.3. Vascular Endothelial Inflammation

Similar to blueberries, strawberries have been implicated in reducing inflammation in vascular endothelial cells. *db*/*db* mice, a mouse model of genetic obesity and type 2 diabetes, were used to assess the impact of strawberry consumption on endothelial cell inflammation [93]. Addition of freeze-dried strawberry powder (2.35% of the diet by weight) reduced monocyte binding and inflammation in aortic vessels in *db*/*db* mice. These changes were accompanied by improved endothelial vasorelaxation and lower blood pressure. The strawberry powder also reduced inflammation in carotid artery endothelial cells. Their evidence suggested that the mechanism by which the strawberries suppressed endothelial inflammation was through reduced oxidative stress, as the berries reduced the expression of Nox2. The reduction in Nox signaling likely decreased NF-κB signaling in the endothelial cells, as evidenced by reduced IKKβ expression. The reduction in IKKβ should lead to less nuclear NFκB. Importantly, addition of the strawberries to the diet did not affect body weight, indicating that these improvements are due directly to strawberry consumption and are not subsequent to weight loss. Furthermore, the amount of freeze-dried strawberries added to the diet can feasibly be consumed by humans (~160 g strawberries per day) [93]

### 4.4. Dysbiosis and Inflammation

Like blackberries and blueberries, strawberries have been shown to alter the composition of the gut microbiota. In a mouse model of colitis, addition of freeze-dried strawberry powder (5% of the diet by weight) increased α diversity of the gut microbiota [94]. This alleviation of gut microbiota dysbiosis was accompanied by decreased production of the proinflammatory cytokines, TNF-α, IL-1β, and IFNγ, in the colonic mucosa. The mechanisms mediating the anti-inflammatory effects of strawberries appeared to be through a reduction in COX-2, iNOS, and NF-κB signaling [94]. While this study was not conducted in a model of obesity-induced inflammation, its implications for obesity-associated inflammation are clear. It has been well established that HFDs and obesity alter the gut microbiota. This study demonstrates that adding strawberries to the diet in an amount that can feasibly be consumed by humans is capable of affecting the gut microbiota and improving inflammation. Direct testing of the hypothesis that strawberries reduce inflammation through alterations in the gut microbiota in a model of obesity is needed.

## 5. Anti-Inflammatory Effects of Raspberries

### 5.1. Obesity-Induced Inflammation

Because of their bioactive components, several studies have been conducted using raspberries to determine whether they have potential in alleviating the elevated inflammatory state of obesity. The results of these studies have been promising.

Supplementation of an HFD with freeze-dried raspberry powder (5% by weight) for 10 weeks reduced HFD-induced inflammation in skeletal muscle in mice [95]. The mechanism by which raspberry reduced skeletal muscle inflammation is through AMPK, as the ability for raspberries to prevent HFD-induced inflammation in the muscle was absent in AMPKα1^−/−^ mice. Inflammatory status of other tissues was not reported. Raspberry supplementation reduced body weight in an AMPKα1-dependent manner, but had no effect on energy intake, implying that perhaps raspberries increased energy expenditure [95]. This was demonstrated in another study showing that supplementation with the pulp fraction of raspberries increased energy expenditure in mice fed a HFD [96]. Similarly, raspberry anthocyanin extracts added to an HFD (200 mg/kg diet for 12 weeks) attenuated body weight gain by 64% in mice without affecting energy intake [97]. In this study, the raspberry-anthocyanin extract reduced markers of oxidative stress (MDA) and inflammation (TNF-α, IL-6, and NF-κB) in the liver [97]. The reduction in hepatic MDA concentrations is likely due to the increases in hepatic GSH and SOD [97].

Supplementation of an HFD with freeze-dried raspberry powder (20% by weight) for 13 weeks reduced body weight but did not affect plasma plasminogen activator inhibitor-1 concentrations or spleen size in mice [35]. No other markers of inflammation were measured. Given the relatively high dose of raspberry powder in the diet, it would not be surprising to see effects on inflammation in other tissues. The anti-inflammatory effects of red raspberry polyphenolic extracts were more robust when extracts from the raspberry pulp portion were obtained when compared to polyphenols extracted from the whole fruit or the seed [96]. Mice were fed an HFD supplemented with polyphenolic extracts from either the whole fruit, the pulp, or the seeds of the raspberry for 16 weeks, ad libitum (120 mg of raspberry polyphenols per kg BW/day). Polyphenolic extracts isolated from the raspberry pulp and whole fruit were most effective at attenuating HFD-induced inflammation, including reducing HFD-induced increases in crown-like structures, inflammatory gene expression, and IκBα degradation in white adipose tissue. This indicates that the mechanism by which raspberry polyphenols reduce inflammation is likely through down-regulation of the NF-κB pathway. Polyphenols isolated from the whole fruit or the pulp also attenuated HFD-induced activation of the NOD-, LRR- and pyrin domain-containing protein 3 (NLRP3) inflammasome in adipose tissue macrophages in vivo. These findings were confirmed in an in vitro model of bone marrow-derived macrophages treated with raspberry polyphenols (10 µg/mL for 12 h). These anti-inflammatory effects were accompanied by a significant improvement in glucose and insulin tolerance in the mice that received polyphenols extracted from the pulp only. Interestingly, only polyphenols extracted from the pulp reduced body weight, and they did so by increasing energy expenditure without affecting energy intake [96]. Despite polyphenols extracted from the raspberry seed not showing robust effects in that study, another study reported that supplementing a high-fat, high-sucrose diet with raspberry seed flour for 12 weeks reduced adipose tissue inflammation and oxidative stress and improved insulin tolerance in mice [98]. These effects were attributed to the high ellagic acid content of the raspberry seed flour (equivalent to 0.03% of ellagic acid).

### 5.2. LPS-Induced Inflammation

LPS is often used as a model to induce an inflammatory response in vivo and in vitro. Given the findings that raspberries exhibit anti-inflammatory properties in models of diet-induced obesity, it was not surprising to learn that raspberries attenuate LPS-induced inflammation. To date, there has been limited work examining the protective effects of berries against neuroinflammation. This is an important area to study, as obesity and consumption of an HFD are known to lead to neuroinflammation. One study examined the effects of bioaccessible raspberry metabolites on neuroinflammation in microglia, the immune cells of the brain [99]. Raspberry extracts were digested in vitro to model the chemical changes that occur during digestion, and physiologically relevant concentrations of raspberry metabolites were used to treat LPS-stimulated microglial cells [99]. Pre-treatment of the microglia with bioaccessible raspberry extracts (1.25 µg of gallic acid equivalents) suppressed the LPS-induced activation of microglia and TNF-α release from the microglia. The ability of these raspberry metabolites to suppress neuroinflammation was not dependent on ROS scavenging mechanisms [99]. Other studies found similar abilities of raspberry extracts to inhibit LPS-induced inflammation. Treating RAW 264.7 macrophages with anthocyanin-rich fractions from raspberries (100, 150, 200 μg/mL) reduced LPS/IFNγ-induced increases in IL-1β and IL-6 expression in a concentration-dependent manner [100]. The mechanism mediating these effects was likely a down-regulation of NF-κB and activator protein 1 (AP-1) signaling, as evidenced by reduced activities of these transcription factors measured by luciferase reporter assays [100].

Black raspberries have also been evaluated for their anti-inflammatory properties in a model of LPS-induced inflammation in RAW 264.7 macrophages [101]. Black raspberry fractions reduced the expression of iNOS, COX-2, and inflammatory cytokines in macrophages. Cyanidin, C3G, and cyanidin-3-rutinoside were identified as the bioactive components most responsible for mediating the anti-inflammatory effects. The mechanisms targeted by the black raspberry fraction appear to be through down-regulation of the MAPK and signal transducer and activator of transcription 3 (STAT3) signaling pathways. The authors concluded that co-administration of these three compounds is more effective than one compound alone [101]. Other studies have also shown that raspberry supplementation reduced STAT3 signaling, albeit in the colonic tissues of mice with chronic colitis (5% of dry feed weight for 10 wk) [102]. Furthermore, raspberry extracts increased expression of catalase and Nrf2 and reduced NF-κB signaling in a skin cell exposed to UVB radiation. While not a model of obesity, these studies are in agreement with other studies demonstrating that Nrf2 and NF-κB are likely mediators of the anti-inflammatory properties of raspberries [103,104]. Together, these data suggest that raspberries have the potential to reduce inflammation in a variety of models. Potential targets of raspberry metabolites include the MAPK, NF-κB, Nrf2, and STAT3 signaling pathways, among others.

## 6. Conclusions

Obesity-associated inflammation is linked to increased risk of chronic diseases, including type 2 diabetes, cardiovascular disease, and cancer. Studies examining consumption of berries in humans showed the efficacy of berries in reducing inflammation and other risk factors for type 2 diabetes and cardiovascular disease. Preclinical rodent and cell culture studies provide robust evidence that berries and their bioactive components have beneficial effects not only on inflammation, but also on biomarkers of many of these chronic diseases (Supplemental Tables S1 and S2). The primary mechanisms mediating the anti-inflammatory effects of berries include a reduction in NF-κB signaling that may be secondary to reduced oxidative stress, a down-regulation of TLR4 signaling, and an increase in Nrf2. Robustly conducted studies also implicate a down-regulation of the NLRP3 inflammasome in adipose tissue macrophages. Berries contain a number of factors that could potentially elicit health benefits including polysaccharides, fiber, vitamins, minerals, polyphenols, and other phytonutrients. Most of the studies included in this review utilized freeze-dried berry powders, making it impossible to pinpoint which bioactive compound(s) is/are responsible for the effects of the berries. Further elucidation of the mechanisms mediating the anti-inflammatory effects of berries has the potential to generate new complementary therapeutics in the treatment of obesity-associated inflammation and related comorbidities.

## Data Availability

Not applicable.

## Supplementary Materials

The following are available online at https://www.mdpi.com/2072-6643/13/2/334/s1, Table S1: Summary of Animal Studies Examining Effects of Berries or their Components on Inflammation, Table S2: Summary of Animal Studies Examining Effects of Berries or their Components on Inflammation.
