# Supplementary material for: Berries as a Treatment for Obesity-Induced Inflammation: Evidence from Preclinical Models
Source: Nutrients. 2021 Jan 23;13(2):334. doi: 10.3390/nu13020334 (PMC7912458; doi:10.3390/nu13020334)
Supplement: Supplementary file 1 [file nutrients-13-00334-s001.pdf]

nutrients

MDPI

Review

Berries as a Treatment for Obesity-Induced Inflammation: Evidence from Preclinical Models

Hannah Land Lail, Rafaela G. Feresin, Dominique Hicks, Blakely Stone, Emily Price and Desiree Wanders *

Table S1. Summary of Animal Studies Examining Effects of Berries or their Components on Inflammation.

Animal Studies				
**Model**	**Treatment**	**Effect on Inflammation**	**Mechanism**	**Reference**
Blackberries				
Male C57BL/6 mice	Anthocyanins from blackberry extract (200 mg/kg diet)	Reduced gene expression of NF-κB, TNF-α, and IL-6 in liver	N/A	[32]
Female SKH-1 hairless mice	Topical application of blackberry extract (10 and 20%)	Reduced expression of iNOS, PGE_2, IL-6, and TNF-α in skin	Reduced NF-κB, iNOS, COX-2, PGE_2 signaling	[40]
Male C57BL/6J mice	Freeze-dried blackberry in diet (20% of the diet)	Null	N/A	[30]
Male C57BL/6J mice	Freeze-dried blackberry supplementation (6.3% of diet in weight; 470 mg C3G equivalents/kg diet)	Null	N/A	[31]
Wistar rats	Freeze-dried blackberry anthocyanin-rich extract (25 mg/kg BW/day)	Attenuated neuroinflammation; Decreased expression of TCK-1 in the hippocampus	Stimulating tryptophan metabolism in the kynurenine pathway	[41]
Male C57BL/6J mice	Fermented blueberry-blackberry beverage	Null	N/A	[33]
Female Sprague–Dawley rats	Freeze-dried blackberry powder (10% of the diet)	Reduced ovariectomy-induced increase in body weight Reduced mRNA expression of NF-κB and COX-2 in the liver	N/A	[34]
Blueberries				
Male C57BL/6 mice	Blueberry anthocyanins (200 mg/kg diet)	Decreased IL-6 and TNF-α in liver; Increased SOD and GPx antioxidant activity in liver and serum	Decreased NF-κB	[32]
C57BL/6 mice	HFD supplemented with freeze-dried blueberry powder (5% or 10% of diet)	Reduced expression of IL-1β, IL-2, IL-7 TNF-α, GM-CSF and MCP-1 Reduction of T-helper 1 cells and Tc cells in the spleen	N/A	[50]
Male C57BL/6 mice	HFD supplemented with freeze-dried blueberry powder (4% of diet)	Reduced TNF-α, MCP-1, and IL-10 expression, and increased GPx3 antioxidant activity in adipose tissue	N/A	[51]
Obese Zucker rats	2% Blueberry diet	Reduced ROS, superoxide, peroxynitrite, IL-1β, IL-18 in the kidney	Reduced TLR4 expression and MAPK phosphorylation, inhibited NF-κB activity; Increased Nrf2 expression	[52]
Male albino rats with STZ-induced diabetes	Blueberry anthocyanins (20, 40, and 80 mg/kg BW/day) for 12 weeks	Increased GSH, GPx activity Decreased MDA, ROS levels in retinal cells Decreased IL-1β in serum	Increased Nrf2 and HO-1	[55]
Male Wistar rats	Freeze-dried blueberry powder (10% of HFD)	Decreased TNF-α and IL-6 in adipose tissue; Decreased MDA expression in the liver	Reduced NF-κB	[76]
Obese male Zucker Rats	Wild blueberry (8% of diet)	Reduced serum IL-6, TNF-α Reduced hepatic NF-κB, IL-6, TNF-α, and CRP Reduced adipose NF-κB, IL-6, and TNF-α	Reduced NF-κB in liver and adipose tissue	[53]
Male C57BL/6J mice	Freeze-dried blueberry supplementation (5% w/w, 470 mg CGE/kg diet)	Null	N/A	[31]
Strawberries				

Male C57BL/6J mice	Freeze-dried whole SB powder (2.35% of diet by weight)	Reduced expression of IκKβ, NOX2, IκBα, NOX4, monocyte binding, blood pressure, and inflammation in CA endothelial cells	Reduced NF-κB	[88]
Male CD-1 mice	Freeze-dried whole SB powder (2.5 or 5% of diet by weight)	Reduced TNF-α, IL-1β, IFN-γ; IL-17, decreased iNOS, p-53;<br>Increased GSH/GSSG ratio<br>Suppressed expressions of cleaved caspase-3 and c-JUN	Reduced NF-κB and COX-2	[89]
Male Wistar rats	P3G-enriched strawberry powder (8 mg P3G/kg/day) for 8 weeks	Reduced inflammatory cell infiltration in liver and left ventricle	N/A	[29]
Male Wistar rats	Freeze-dried SB/BB powder (5:1) (6% of diet by weight) for 6 weeks	Reduced circulating MCP-1	N/A	[81]
C57BL/6J mice	2.6% SB powder (equivalent to 1 human serving)	Reduced circulating CRP	N/A	[82]
		Raspberries		
Wild-type (WT) and AMPKα1$^{-/-}$ mice	HFD plus raspberry (5% HFD dry weight)	Reduced TNF-α, IL-1β, IL-6, IL-8 in skeletal muscle	Reduced of NF-κB/p65; dependent on AMPKα1	[90]
Male C57BL/6 mice	HFD or HFD supplemented with a polyphenolic fraction from whole fruit, pulp, or seed.	Decreased the recruitment of macrophages and adipocyte hypertrophy in adipose tissue	Inhibited NLRP3 inflammasome activation	[91]
Male C57BL/6 mice	HFHS diet supplemented with ellagic acid from raspberry seed flour	Reduced IL-6, IL-8, F4/80, TNFα, and MCP-1 expression	N/A	[93]
C57BL/6 mice	HFD supplemented with raspberry anthocyanins (200 mg/kg diet/day) for 12 weeks	Reduced MDA, TNF-α, and IL-6 in the liver	Elevated SOD, GSH activities; down-regulated NF-κB	[92]

Table S2. Summary of cell culture studies examining effects of berries or their components on inflammation.

Cell Culture Studies				
**Model**	**Treatment**	**Effect on Inflammation**	**Mechanism**	**Reference**
		Blackberries		
RAW 264.7 Macrophages	Anthocyanin- and proanthocyanin- enriched fractions from blueberry-blackberry wine blends (50 and 100 µM)	Reduced expression of iNOS, NO, and COX-2	Reduced NF-κB signaling	[28]
RAW 264.7 Macrophages	Anthocyanin-enriched fractions of blueberry-blackberry wine blends	Reduced secretion of NO and TNF-α	Reduced NF-κB signaling	[39]
RAW 264.7 Macrophages	Blackberry anthocyanins (0-20 µg/mL)	Reduced IL-1β and TNFα mRNA expression	Inhibition of NF-κB translocation	[38]
		Blueberries		
Human Retinal Capillary Endothelial cells (HRCECs)	Blueberry anthocyanin extract (BAE) (Malvidin Malvidin-3-glucoside Malvidin-3-galactoside)	Decreased ROS	Increased catalase, superoxide dismutase Downregulated Nox4 Decreased VEGF Inhibited Akt pathway	[56]
Human Retinal Pigment Epithelial cells	Blueberry anthocyanin extract (BAE) (Malvidin Malvidin-3-glucoside Malvidin-3-galactoside)	Inhibited apoptosis Decreased VEGF Altered Akt pathway	N/A	[57]
Human Aortic Endothelial cells (HAECs)	Blueberry metabolites (700nM benzoic acid-4-sulfate, 5µM hippuric acid, 3µM hydroxyhippuric acid, 75nM isovanillic acid-3-sulfate, and 75nM vanillic acid-4-sulfate)	Reduced monocyte adhesion Reduced inflammatory markers IL-8, MCP1, and VCAM-1	N/A	[69]
Human Umbilical Vein Endothelial cells (HU-VECs)	Anthocyanin-rich- fraction from blueberries (from 0.01 to 10 µg/mL)	Reduced THP-1 adhesion	Reduced monocyte adhesion of THP-1 to HUVECs by decreasing E-selectin concen-trations	[70]
Human Aortic Endothelial cells (HAECs)	Blueberry metabolites (700nM benzoic acid-4-sulfate, 5µM hippuric acid, 3µM hydroxyhippuric acid, 75nM isovanillic acid-3-sulfate, and 75nM vanillic acid-4-sulfate Or anthocyanins: malvidin-3-glucoside and cyanidin-3-glucoside)	Reversed the effects of palmitate by decreasing ROS, Nox4, chemokines, ICAM-1, IκBα, mono-cyte adhesion, and NO production	N/A	[71]
Human Umbilical Vein Endothelial cells	Blueberry anthocyanin extract (BAE) (Malvidin Malvidin-3-glucoside Malvidin-3-galactoside at 1, 10, 50, and 100µM)	Decreased MCP1, ICAM-1, and VCAM-1 expres-sion Decreased IkBα degradation and altered nuclear translocation of P65	Inhibited NF-κB pathway	[72]
		Strawberries		
RAW 264.7 Macrophages	Sheolhyang strawberry fraction	Reduced TNF-*a*, IL-1β, and iNOS expressions	Blocked degradation of IκBα Inhibited NF-κB and MAPK signaling	[86]
Human Dermal Fibroblasts (HDF)	Alba strawberry methanolic extracts (25–1000 µg/mL)	Reduced ROS/NO	N/A	[85]

RAW 264.7 Macrophages	SB extract (100 µg/mL)	Reduced LPS-induced ROS, TNF-α, IL-1β, and IL-6, pIκβ*a*	Increased Nrf2; reduced NF-κB; increased AMPK-activation	[87]
		Raspberries		
RAW 264.7 Macrophages	Red raspberry extract (100, 150 and 200 µg/mL)	Attenuated LPS/IFN-γ-induced inflammatory responses in RAW 264.7 cells	Blocked activation of NF-κB and MAPK/JNK	[95]
Murine Macrophages	Black raspberry fractions	Suppressed mRNA and protein expressions of TNF-α, IL-6, and IL-1β	Inhibited MAPKs and STAT3	[96]
Microglia cells	Raspberries subjected to *in vitro* digestion	Inhibited microglial proinflammatory activation by LPS; decreased Iba1 expression, TNF-α release, and NO production	N/A	[94]
